# The Effect of Gabapentin on Intensity and Duration of Hot Flashes in Postmenopausal Women: A Randomized Controlled Trial

**DOI:** 10.5539/gjhs.v5n6p126

**Published:** 2013-09-10

**Authors:** Najmieh Saadati, Razieh Mohammad Jafari, Solmaz Natanj, Parvin Abedi

**Affiliations:** 1 Fertility Infertility and Perinatology Research Center, Ahvaz Jundishapur University of Medical Sciences, Ahvaz, Iran; 2 Menopause Andropause Research Center, Ahvaz Jundishapur University of Medical Sciences, Ahvaz, Iran

**Keywords:** menopause, gabapentin, hot flash, intensity, duration

## Abstract

**Background:**

Menopause is the stage of time in which the menstruation stops following the loss of ovarian activity. The purpose of this study was to find out the effectiveness of gabapentin on hot flashes in postmenopausal women.

**Materials and Methods:**

A randomized controlled trial from Feb 2010 to 2011 was conducted. Sixty postmenopausal women who were referred to obstetrics and gynecology ward of two educational hospitals were recruited and divided into two groups (intervention and control). Intervention group received 300 mg gabapentin three times a day for three months, while control group received placebo. The Intensity and duration of hot flashes in women scored and recorded using visual analog scale. Independent, Paired t-test and chi-square test were used for analyzing data.

**Results:**

Intensity of hot flashes in the beginning of research in the intervention group was significantly different with the first, second and third follow-up visit (P<0.05). Also at the end of intervention a significant difference between intervention and control groups were observed regarding the intensity, frequency and duration of hot flashes (P<0.05 and P=0.01 respectively).

**Conclusion:**

According to the findings of this study; it appears that the use of gabapentin could decrease the intensity, duration and frequency of hot flashes in postmenopausal women. For postmenopausal women who hormone therapy is contraindicated, gabapentine could be an acceptable alternative.

## 1. Introduction

Menopause is the stage of time in which the menstruation stops following the loss of ovarian activity. Determining the average age of menopause is somewhat difficult. According to studies, it is estimated that the average age of menopause is 50-52 years (McKinlay et al., 1985). Short term changes in the postmenopausal vasomotor symptoms are hot flashes and night sweats. Approximately 50-80% of the women older than 45 years experience flashing. Vasomotor symptoms are common in the United States. About 50% of postmenopausal women suffer from hot flashes and about 40% of them have night sweats (Alexander & Moore, 2007). The most common symptom of menopause is hot flash that often disappears after years, but in 15-20% of women leaves adverse impact on their lives (Martin & Manson, 2008). Flashing frequency may be too low or too much. Frequency and intensity of hot flashes are more at night and stresses. The frequency, intensity and duration of hot flashes are lower in cool rather than warm climates (Cope et al., 2005). While hormone replacement therapy (HRT) can eliminate hot flashes (Creasman, 2002; O’Meara et al., 2004) the treatment should not last more than 4-5 years, because of the risk of breast cancer increases in long-term using of HRT (Alexander & Moore, 2007). Gabapentin is a suitable alternative at a time when HRT is contraindicated (Brown & Wright, 2009). In a study conducted in the Mayo Clinic, patients suffered from hot flashes received gabapentin, and 66% of patients had a significant reduction in hot flash’s frequency at the end of 4th week. The severity of hot flashes had 70% reduction. Also, all patients who completed four weeks of treatment, showed a significant improvement in other symptoms (Loprinzi et al., 2002). There is lack of enough information about using gabapentin for reducing hot flashes; therefore this study was designed to assess the effect of gabapentin on intensity and duration of hot flashes in post menopausal women.

## 2. Materials and Methods

### 2.1 Study Design and Population

In this randomized controlled trial, 60 postmenopausal women who were referred to two educational hospitals (Imam Khomeini and Razi) from Feb 2010 to 2011 were enrolled. Women were randomly divided into two groups (intervention and control groups) with 30 participants in each group. The study was approved by the Ethics Committee of Ahvaz Jundishapur University of Medical Sciences, and written informed consent was obtained from all women. The inclusion criteria were; women who experienced menopause at least one year ago and natural had menopause. Women who were under the hormone replacement therapy and with known chronic diseases were excluded from study. Women were assessed for the intensity, frequency and duration of hot flashes before and three months after intervention each month. The intensity of hot flashes was measured by visual analog scale and the frequency and duration were recorded in a checklist. Women with moderate and severe hot flashes in terms of intensity were selected and randomly classified in the gabapentin or placebo groups.

### 2.2 Intervention

Intervention group received 300 mg gabapentin three times a day for a period of three months (Tehran Darou Co, Tehran, Iran), while control group treated with placebo. Both intervention and control groups have been visited four times with the frequency of one month. The intensity, duration and number of hot flashes in women scored and recorded using visual analog scale and a checklist.

### 2.3 Statistical Analysis

Data were analyzed using SPSS 17.0. The descriptive statistics were utilized to describe two groups. The differences in hot flashes before and after intervention and also between groups were compared using independent t-test, chi-square and paired t-test. The p-value less than 0.05 considered as significant.

## 3. Results

**Figure 1:**
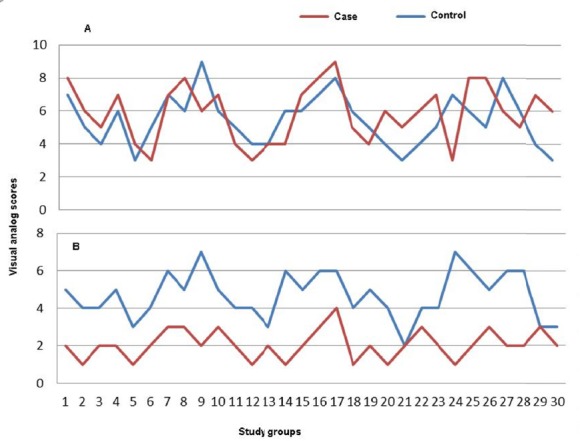
A) Intensity of hot flashes in the beginning of the study B) The intensity of hot flashes at the end of the study in two intervention and control groups

The mean age of intervention and control groups was 51.43 and 50.96 years, respectively. Socio-demographic characteristics of participants are listed in Table 1. At the end of treatment a significant difference was observed in the intensity and duration of hot flashes between intervention and control groups (P = 0.001) (Table 2 and Figure 1). The intervention group was significantly more satisfied with treatment than that in the control group (P = 0.02). The most common drug side effect which observed in first, second and third visits was drowsiness (13.3%). In intervention and control groups hot flashes reduction base on hot-flash score at baseline, end of first, second and third month was reported as follow: in the intervention group 58.6%, 45%, 32.3% and 20.6% and in the control group was, 54.6%, 49%, 48.3% and 47%. There was significant difference between the mean of hot flashes’ intensity in the intervention and control groups and also in the beginning of treatment and the end of third month in the intervention group (P <0.05).

**Table 1:** Socio-demographic characteristics of participants in the Gabapentin and control groups

Variables	Gabapentin	Control	P value

	Mean± SD or N (%)		
**Age**	51.4±2.1	50.9±2.1	0.39
**Menopause age**	50.8±2.07	50.4±2.01	0.32
**Menarche age**	11.9±1	12.2±1	0.45
**Intensity of hot flashes**	5.8±1.7	5.4±1.5	0.24
**Duration of menopause (Months)**	16.9±2.2	17.06±2.4	0.44
**Dysparunia**	17 (56.6)	19(63.3)	0.35

**Table 2:** The mean of intensity, duration and number of hot flashes in the beginning, 1^st^, 2^nd^ and 3^rd^ month after intervention in the gabapentin and control groups

Variables	Gabapentin	Control	P value

	Mean± SD or N(%)		
**Intensity**			
Before intervention	5.8±1.7	5.4±1.5	0.34
1^st^ month	4.5±1.5	4.9±1.3	0.21
2^nd^ month	3.2±1.04	4.8±1.3	<0.001
3^rd^ month	2.06±0.78	4.7±1.2	<0.001
**Duration (min)**			
Before intervention	2.8±1.87	2.6±1.4	0.64
1^st^ month	2.4±1.1	2.5±1.3	0.065
2^nd^ month	1.90±1.03	2.4±1.2	0.02
3^rd^ month	0.91±1.31	2.2±1.4	<0.001
**Number of hot flashes per week**			
Before intervention	13.06±4.4	13.03±4.6	0.97
3^rd^ month after intervention	4.2±4.8	13.06±4.3	<0.001

## 4. Discussion

Estrogen treats flashing by increasing the sweating threshold, although the underlying mechanisms are not fully known (Freedman et al, 1995). Collected data suggest that treatment with estrogen for years after the start of menopause associates with a high risk of coronary disease, whereas initiating treatment at a beginning of menopause, eliminate this risk. Recently the public opinion is that the short-term treatment with minimum dose of estrogen is acceptable treatment in women who have moderate to severe symptoms with healthy heart (Alexander & Moore, 2007). It is shown that the risk of fatal ovarian cancer with long-term use of estrogen will increase. In women who HRT is contraindicated, alternative non-hormonal treatments are preferred (Rozenberg et al., 2013). Gabapentin is an antiepileptic drug that the Food and Drug Administration of America introduce it as a treatment for partial epilepsy and postherpetic neurology. Drowsiness, dizziness, ataxia, tiredness, nystagmus, and peripheral edema are some side effects of this drug in the treatment of partial epilepsy and postherpetic neurology (Brown & Wright, 2009). Although there are few researches about this drug, but they are indicating that; it is safe and effective in treating hot flash. The dose used in hot flash is completely tolerated and it’s most common side effects is drowsiness as same as in our study (Brown & Wright, 2009). In the present study, the intensity of hot flashes in the intervention group reduced more than 50% after three months in the intervention group. In a study hot flash reduction in gabapentin group was 51% and in placebo group was 26% compared to the pre-treatment by the end of the 4th week (Butt et al., 2008). In another study by Pandaya et al. (2004), 22 patients who suffered from breast cancer treated with tamoxifen, and had at least two hot flashes per day, treated with 300 mg of gabapentin three times daily for a period of four weeks. Sixty patients who completed their treatment had a modest reduction in hot flashes duration by 73.6% and frequency by 44.2% and its severity by 52.6%.

In a study by Pandaya et al. (2005), on 420 women with breast cancer that experienced two or more than two hot flashes per day, results showed that women who treated with 900mg/day gabapentin for 8 weeks responded to the treatment better than those received 300mg/day. A systematic review that was conducted on 7 trials on 901 patients between 2002 and 2008 showed that; total doses of gabapentin performed was from 900 to 2400mg/day and there was a significant reduction in both the frequency of hot flashes (weighted mean difference= 23.72, CI, 16.46-30.97, p<0.001) (Toulis et al., 2009).

In another study in which 59 postmenopausal women received either 900 mg gabapentin or placebo for 12 weeks, results showed that; gabapentin could reduce the frequency of hat flashes by 45% and 54% reduction in hot flash composite score from baseline. When women received higher dose of gabapentin (2700mg per day), hat flashes reduced by 54% and 67% in frequency and composite score from baseline (Guttuso et al., 2003). Our results are not in line with Guttuso et al. (2003), when gabapentin with dosage of 900 mg per day could reduce more than 50% reduction in frequency of hot flashes while in the study, hot flashes reduced efficiently when the researchers increased the dose of gabapentin. This discrepancy between the present study and Guttuso et al. (2003), may result from that; they recruited women with severe hot flashes, while in our study we recruited moderate and sever hot flashes.

In a study by Tavassoli et al. (2009), in Iran, researchers found that 8 weeks treatment with gabapentin could reduced the frequency of hot flashes by 56% and composite score (frequency and severity combined into one score) by 50%. These results are in line with the present study with one exception; that women in our study received gabapentin for 12 weeks, while in the Tavassoli et al. (2009), women followed-up for 8 weeks.

This is the first time that we conducted a study on the effect of gabapentin on hot flashes in postmenopausal women with duration of 12 weeks in Iran. Further researches with comparing this drug with HRT will recommended.

## 5. Conclusion

According to the findings of this study it appears that the use of gabapentin in the treatment of hot flashes will be useful. Further studies for comparing the effect of gabapentin with HRT to reduce hot flashes in postmenopausal women are recommended.

## References

[ref1] Alexander I. M, Moore A (2007). Treating vasomotor symptoms of menopause: The nurse practitioner’s perspective. J Am Acad Nurse Pract.

[ref2] Brown J. N, Wright B. R (2009). Use of gabapentin in patients experiencing hot flashes. Pharmacotherapy.

[ref3] Butt D. A, Lock M, Lewis J. E, Ross S, Moineddin R (2008). Gabapentin for the treatment of menopausal hot flashes: a randomized controlled trial. Menopause.

[ref4] Creasman W. T (2002). Estrogen and cancer. Gynecol Oncol.

[ref5] Freedman R. R, Norton D, Woodward S, Cornélissen G (1995). Core body temperature and circadian rhythm of hot flashes in menopausal women. J Clin Endocrinol Metab.

[ref6] Guttuso J. R. T, Kurlan R, McDermott M. P, Kieburtz K (2003). Gabapentin’s effects on hot flashes in postmenopausal women: a randomized controlled trial. Obstetrics & Gynecology.

[ref7] Loprinzi L, Barton D. L, Sloan J. A, Zahasky K. M, Smith D. A, Pruthi S, Christensen B. J (2002). Pilot evaluation of gabapentin for treating hot flashes. Mayo Clin Proc.

[ref8] Martin K. A, Manson J. E (2008). Approach to the patient with menopausal symptoms Menopause. J-Clin-Endocrinal-Metab.

[ref9] McKinlay S. M, Bigano N. L, McKilay J. B (1985). Smoking and age at menopause. Ann Intern Med.

[ref10] O’Meara E. S, Rossing M. A, Daling J. R, Elmore J. G, Barlow W. E, Weiss N. S (2004). Hormone replacement therapy after a diagnosis of breast cancer in relation to recurrence and mortality. J Natl Cancer Inst.

[ref11] Pandya K. J, Morrow G, Roscoe J. A, Zhao H, Hickok J. T, Pajon E, Flynn P. J (2005). Gabapentin for hot flashes in 420 women with breast cancer: a randomized double- blind placebo controlled trial. Lancet.

[ref12] Pandya K. J, Thummala A. R, Griggs J. J, Rosenblatt J. D, Sahasrabudhe D. M, Guttuso T. J, Roscoe J. A (2004). Pilot study using gabapentin for tamoxifen-induced hot flashes in women with breast cancer. Breast Cancer Research and Treatment.

[ref13] Rozenberg S, Vandromme J, Antoine C (2013). Postmenopausal hormone therapy: risks and benefits. Nat. Rev. Endocrinol.

[ref14] Tavassoli F, Sharifian Attar J, Ghomian N, Talebi M, AfzalAghaei M (2009). Effect of gabapentin on reduction of hot flash in menopausal women. Birjand University of Medical Sciences.

[ref15] Toulis K. A, Tzelllos T, Kouvelas D, Goulis D. G (2009). Gabapentin for the treatment of hot flashes in women with natural of tamoxifen- induced menopause: A systematic review and meta-analysis. Clinical Therapeutics.

